# Reciprocal White Matter Changes Associated With Copy Number Variation at 15q11.2 BP1-BP2: A Diffusion Tensor Imaging Study

**DOI:** 10.1016/j.biopsych.2018.11.004

**Published:** 2019-04-01

**Authors:** Ana I. Silva, Magnus O. Ulfarsson, Hreinn Stefansson, Omar Gustafsson, G. Bragi Walters, David E.J. Linden, Lawrence S. Wilkinson, Mark Drakesmith, Michael J. Owen, Jeremy Hall, Kari Stefansson

**Affiliations:** a Cardiff University Brain Research Imaging Centre, School of Psychology, Cardiff, United Kingdom; b Neuroscience and Mental Health Research Institute, Cardiff, United Kingdom; c Division of Psychological Medicine and Clinical Neurosciences, Cardiff, United Kingdom; d MRC Centre for Neuropsychiatric Genetics and Genomics, School of Medicine, Cardiff University, Cardiff, United Kingdom; e deCODE genetics/Amgen, Reykjavik, Iceland; f Faculty of Electrical Engineering, Reykjavik, Iceland; g Faculty of Medicine, University of Iceland, Reykjavik, Iceland

**Keywords:** 15q11.2 BP1-BP2, Copy number variant, CYFIP1, Diffusion tensor imaging, Fragile X syndrome, Genetics

## Abstract

**Background:**

The 15q11.2 BP1-BP2 cytogenetic region has been associated with learning and motor delays, autism, and schizophrenia. This region includes a gene that codes for the cytoplasmic FMR1 interacting protein 1 (*CYFIP1*). The CYFIP1 protein is involved in actin cytoskeletal dynamics and interacts with the fragile X mental retardation protein. Absence of fragile X mental retardation protein causes fragile X syndrome. Because abnormal white matter microstructure has been reported in both fragile X syndrome and psychiatric disorders, we looked at the impact of 15q11.2 BP1-BP2 dosage on white matter microstructure.

**Methods:**

Combining a brain-wide voxel-based approach and a regional-based analysis, we analyzed diffusion tensor imaging data from healthy individuals with the deletion (*n =* 30), healthy individuals with the reciprocal duplication (*n =* 27), and IQ-matched control subjects with no large copy number variants (*n =* 19), recruited from a large genotyped population sample.

**Results:**

We found global mirror effects (deletion > control > duplication) on fractional anisotropy. The deletion group showed widespread increased fractional anisotropy when compared with duplication. Regional analyses revealed a greater effect size in the posterior limb of the internal capsule and a tendency for decreased fractional anisotropy in duplication.

**Conclusions:**

These results show a reciprocal effect of 15q11.2 BP1-BP2 on white matter microstructure, suggesting that reciprocal chromosomal imbalances may lead to opposite changes in brain structure. Findings in the deletion overlap with previous white matter differences reported in fragile X syndrome patients, suggesting common pathogenic mechanisms derived from disruptions of cytoplasmic CYFIP1-fragile X mental retardation protein complexes. Our data begin to identify specific components of the 15q11.2 BP1-BP2 phenotype and neurobiological mechanisms of potential relevance to the increased risk for disorder.

Copy number variants (CNVs) are rare structural variations of the genome arising from unbalanced meiotic rearrangements that can result in carriers possessing a deletion or duplication of parts of one of the chromosome pairs. An increased burden of CNVs has been observed in several neurodevelopmental and psychiatric diseases, including autism spectrum disorder (ASD), attention-deficit/hyperactivity disorder (ADHD), intellectual disability, and schizophrenia 1, 2. How these damaging variants modify risk for psychopathology is still not well understood at the mechanistic level, but given their relatively high penetrance and cross-disorder pleiotropic effects, significant impact on brain structure and function is anticipated.

Altered white matter (WM) structure has been consistently reported in psychiatric disorders. For instance, in the case of schizophrenia, neuroimaging studies have shown abnormal structural and functional connectivity at both microscopic and macroscopic levels, and such data have been central in supporting various “dysconnectivity” hypotheses of mental disease 3, 4. It follows that a key question for neurobiological research is whether CNVs that are associated with neurodevelopmental disorders, including schizophrenia, are also associated with changes in WM and brain connectivity.

The 15q11.2 BP1-BP2 cytogenetic microdeletion is emerging as a recognized syndrome and has been associated with developmental, speech, language, and motor delays 5, 6, and also with increased susceptibility for epilepsy (7), ADHD (5), ASD (8), and schizophrenia (9). Moreover, recent ultra high-resolution chromosomal microarray analyses report the 15q11.2 BP1-BP2 deletion as the most frequent finding in those with only ASD or with ASD combined with intellectual disability and congenital anomalies (10). The reciprocal duplication has also been associated with increased risk for ASD (11), although its significance is still unclear (8).

Not all the individuals with the BP1-BP2 microdeletion/microduplication are clinically affected, and the genes in this region have variable expressivity. Yet, healthy individuals with the deletion and without a current clinical diagnosis frequently report mild-to-moderate impairments in motor function and deficits across several cognitive domains, including an increased incidence of difficulties in mathematics and reading skills 11, 12, while healthy individuals with the duplication perform to a similar level as population control subjects (12). In a recent study by Ulfarsson *et al.* (13), these cognitive deficits were shown to be accompanied by structural changes in the brain, as assessed by structural magnetic resonance imaging (MRI), in individuals with a deletion or duplication showing reciprocal structural effects, as well as by different patterns of brain activation in tests of reading and mathematics. However, the effect on WM microstructure cannot be assessed with standard MRI, and diffusion tensor imaging (DTI) studies are needed.

The 15q11.2 BP1-BP2 region is adjacent to the areas affected in the Prader-Willi and Angelman syndromes, conditions resulting from deletions of the BP1-BP3 (type I) or the BP2–BP3 (type II) regions at 15q11.2, with the BP1-BP2 deletion partly overlapping the type I but not type II Prader-Willi/Angelman region. Individuals with type I deletion report more severe neurodevelopmental disturbances compared with individuals with the smaller type II deletion 14, 15. The isolated BP1-BP2 region spans ∼500 kb and encompasses four different genes: nonimprinted in Prader-Willi/Angelman syndrome 1 gene (*NIPA1*), nonimprinted in Prader-Willi/Angelman syndrome 2 gene (*NIPA2*), cytoplasmic FMR1 interacting protein 1 (*CYFIP1*), and tubulin gamma complex associated protein 5 gene (*TUBGCP5*) (16). The four genes probably play a role in brain development and function, and some work has been done to understand the extent and mechanism through which they contribute to increased risk for psychiatric disorder in the 15q11.2 BP1-BP2 region (5). For instance, the *NIPA1* gene is known to mediate Mg^2+^ transport and was associated with autosomal dominant hereditary spastic paraplegia (17), which might be caused by abnormal bone morphogenic protein (BMP) signaling as a result of dysregulations in *NIPA1* (18). The *NIPA2* gene encodes for proteins used in renal Mg^2+^ transport and metabolism and, when mutated, can cause childhood absence epilepsy (19). *TUBGCP5* is highly expressed in the subthalamic nuclei, a region linked to obsessive-compulsive disorder and ADHD (20). More is known about the *CYFIP1* gene, which is considered a prominent candidate gene contributing to 15q11.2 BP1-BP2 brain and psychological phenotypes (21). Haploinsufficiency of *Cyfip1* in mouse models, recapitulating the predicted low dosage of *CYFIP1* in 15q11.2 BP1-BP2 microdeletion, has been shown to impact two main processes: 1) the regulation of cytoskeleton remodeling by the binding of CYFIP1 protein to RAC1, and subsequent activation of the WAVE regulatory complex neurons 22, 23; and 2) via direct interaction of the CYFIP1 protein with fragile X mental retardation protein (FMRP), the repression of eIF4E-mediated translation of FMRP target messenger RNAs (24). These actions of CYFIP1 protein in the brain have the potential to influence WM, the former through effects on neuronal structure and integrity and the latter via interactions with FMRP, mutations that are known to be associated with changes in WM structure (25). Loss of FMRP function, due to an expansion repeat in the *FMR1* gene on the long arm of the X chromosome, is a cause of fragile X syndrome (FXS), the most common monogenic form of inherited intellectual disability (26).

Recently, two studies 27, 28 used DTI to investigate differences in WM microstructure, comparing subjects with FXS with subjects without FXS but with similar IQ and levels of autistic symptoms (minimizing confounding effects owing to intellectual ability), and found increased fractional anisotropy (FA) as well as decreased radial diffusivity (RD) and mean diffusivity (MD) in several WM tracts in FXS subjects. Therefore, it might be anticipated, given the close molecular links between CYFIP1 and FMRP, that some degree of phenotypic overlap may be present in FXS and 15q11.2 BP1-BP2 deletion.

In the present work, we employed a DTI approach to assess WM microstructural changes associated with the 15q11.2 BP1-BP2 region in an adult cohort, selected from the Icelandic population, without a known diagnosis of schizophrenia or autism, thereby potentially avoiding the confounding effects of the disorders clinical signs. Combining brain-wide voxel-based approach (FSL Tract-Based Spatial Statistics [TBSS]) with an atlas-based analysis, allowing quantification of the magnitude of regional changes, we hypothesized that we would see a similar pattern of effects as reported for FXS: increased FA in 15q11.2 BP1-BP2 deletion. We also assessed healthy adults with the reciprocal duplication to evaluate the extent of any reciprocal effects on the neural phenotype. Our data begin to identify specific components of the 15q11.2 BP1-BP2 phenotype and mechanisms of potential relevance to the increased risk for disorder.

## Methods and Materials

### Participants

In total, 30 individuals with the 15q11.2 BP1-BP2 deletion, 27 with the reciprocal duplication, and 19 control subjects with no large CNVs (NoCNV) were recruited from a large genotyped sample of approximately 160,000 subjects representing half of the Icelandic population, in which none of the subjects had any other large CNVs. Subjects between 21 and 66 years of age were included in this study, and the number of female and male subjects was the same (38 men and 38 women) and balanced in each condition group. All the subjects were clinically healthy, such that subjects were excluded if they had ICD-10 or DSM-IV diagnoses for schizophrenia or schizoaffective or bipolar disorder; were diagnosed with autism, intellectual disability, or developmental delay at the State Diagnostic and Counselling Centre of Iceland (serves children and adolescents with a disability); met psychoses criteria on the Mini-International Neuropsychiatric Interview (29); were diagnosed with schizophrenia, schizoaffective or bipolar disorder, autism, intellectual disability, or developmental delay according to self-reports (or reports from parents); or were using antipsychotic medication. Approval for this study was obtained from the National Bioethics Committee of Iceland and the Icelandic Data Protection Authority.

The IQ scores were assessed using an Icelandic version of the Wechsler Adult Intelligence Scale 30, 31 including four subtests (vocabulary, similarities, block design, and matrix reasoning). Further details on how these individuals were genotyped and on how the cognitive assessment was performed can be found in Stefansson *et al.*’s (12) study. There were no significant differences in the IQs between groups. Although all the individuals with the deletion were tested, only 11 of 19 from the NoCNV group and 26 of 27 from the duplication group were tested. Demographic information is described in Table 1, and family relationships between subjects are described in Supplemental Table S2.

**Table 1:** Subject Characteristics

Group	Age, Years	Male/Female	IQ Score^a^	Subjects (*N* = 76)
Deletion	42.83 ± 12.5 (21–65)	14/16	101.2 ± 13.8	30
NoCNV	38.95 ± 10.56 (22–59)	12/7	108.3 ± 16.9	19
Duplication	43.48 ± 13.51 (22–66)	12/15	100.8 ± 11.8	27

Values are mean ± SD (range) or *n*. The IQ score included four subtests (vocabulary, similarities, block design, and matrix reasoning).

NoCNV, no large copy number variants.

a Icelandic version of the Wechsler Abbreviated Scale of Intelligence (12). The test was performed in all individuals with the deletion, in 11 of 19 individuals in the NoCNV group, and in 26 of 27 individuals with the duplication.

### Diffusion Tensor Imaging

Water diffusion is anisotropic in healthy nerve fibers, diffusing freely along the fiber tracts but restricted in the perpendicular direction (32). DTI is sensitive to these anisotropic changes, which makes this technique particularly useful for evaluating WM microstructure (33). DTI findings are commonly reported in terms of scalars such as FA, axial diffusivity (AD), RD, and MD.

### Diffusion MRI Acquisition and Preprocessing

MRI data were acquired on a Philips Achieva 1.5T system (Phillips Healthcare, Eindhoven, the Netherlands). A diffusion-weighted echo-planar imaging sequence with sensitivity encoding acceleration was used. Seventeen noncolinear gradient diffusion-weighted images (DWIs) at b = 800 s/mm^2^ and one nonweighted (b = 0 s/mm^2^) image were acquired with the following parameters: echo time = 72 ms, repetition time = 9024 ms, 60 slices, slice thickness = 2 mm, field of view = 240 × 240 mm, acquisition matrix = 144 × 144, resulting in data acquired with a 1.67 × 1.67 × 2 mm voxel resolution.

Diffusion-weighted data were preprocessed using ExploreDTI v.4.8.3 (34) in MATLAB R2015a (The MathWorks, Inc., Natick, MA). First, the Brain Extraction Tool (35) (http://www.fmrib.ox.ac.uk/fsl/) was used to remove nonbrain tissue. Within the ExploreDTI pipeline, eddy currents and head motion correction was performed using an affine registration to the non–diffusion-weighted B_0_ images, with appropriate rotation of the encoding vectors (36). Field inhomogeneities were corrected using the approach of Wu *et al.* (37). Each DWI was nonlinearly warped to the T_1_-weighted image using the FA maps from the DWIs as a reference. Warps were computed using Elastix (38), by using normalized mutual information as the cost function and constraining deformations to the phase-encoding direction. The corrected DWIs were therefore transformed to the same (undistorted) space as the T1-weighted structural images. ExploreDTI was used to generate whole-brain maps of FA, AD, RD, and MD.

### TBSS Analysis of DTI

The corrected FA, AD, RD, and MD maps were analyzed using the FSL’s TBSS tool. TBSS is a whole-brain analysis (39) that starts with a nonlinear registration of the FA maps to a standard FA template (FMRIB58_FA, FMRIB Software Library FA adult template). Then, FA maps are thinned and averaged to create a study-specific WM skeleton template, and the registered FA maps are aligned to this template. An optimal FA threshold of 0.2 was chosen for the binary skeleton mask. Afterward, all the AD, RD, and MD maps were also registered to the FMRIB58_FA template.

General linear models were created to investigate copy number effects at 15q11.2 BP1-BP2. Statistically significant differences were first assessed with a multiple regression model (duplication > NoCNV > deletion and deletion > NoCNV > duplication). Total intracranial volume, age, and sex were included as covariates of no interest. Differences in DTI measures between groups were assessed using voxelwise independent *t* tests (deletion vs. NoCNV, duplication vs. NoCNV, and deletion vs. duplication), in which six different contrasts were used to assess group differences (Table 2). The randomize function from FSL was used with the threshold-free cluster enhancement approach (40), generating cluster-size statistics based on 5000 random permutations to calculate probabilities corrected for multiple comparisons. Significant results were considered with a corrected *p* value <.05 (*p <* .025 for each tail of the two-tailed test). Anatomical WM regions showing significant group differences were identified with the John Hopkins University WM atlas (ICBM-DTI-81) (41).

**Table 2:** Summary of Between-Group FSL Tract-Based Spatial Statistics Analyses Results

Contrast	Whole-Group Analysis			
	FA	AD	RD	MD
Del > NoCNV	**+**	−	−	−
NoCNV > Del	−	−	−	−
NoCNV > Dup	−	−	−	−
Dup > NoCNV	−	**+**	−	−
Del > Dup	**++**	−	−	−
Dup > Del	−	**+**	**++**	**+**

Significant voxelwise comparisons (*p <* .05) are indicated by a plus sign (+) (less significant) or two plus signs (++) (more significant), and nonsignificant results (*p* > .05) are indicated by a minus sign (−). All the *p* values were corrected using the threshold-free cluster enhancement algorithm in FSL Tract-Based Spatial Statistics.

AD, axial diffusivity; Del, deletion; Dup, duplication; FA, fractional anisotropy; MD, mean diffusivity; NoCNV, no large copy number variants; RD, radial diffusivity.

### Regional DTI Metrics Statistical Analyses

Region values of FA, AD, RD, and MD were obtained by averaging over the intersecting voxels between the WM DTI maps with the John Hopkins University WM atlas (ICBM-DTI-81), which comprises 48 tracts (41). To investigate between-group regional differences in FA, AD, RD, and MD, linear regression analysis was performed for each DTI measure and for each WM tract, regressing out age, sex, and total intracranial volume as covariates of no interest. For these, RStudio version 1.1.463 (R Foundation for Statistical Computing, Vienna, Austria) was used to test differences between groups.

To account for multiple testing in the pairwise comparisons, we used the standard false discovery rate method based on the Benjamini-Hochberg approach (42), taking into account the relation between different WM tracts and between DTI metrics. Only significant false discovery rate–adjusted *p* values are reported. Cohen’s *d* effect sizes were calculated for differences between the deletion and duplication groups (43). An interaction between sex and 15q11.2 BP1-BP2 dosage was also evaluated.

## Results

### Between-Group TBSS Analysis

**Figure 1:**
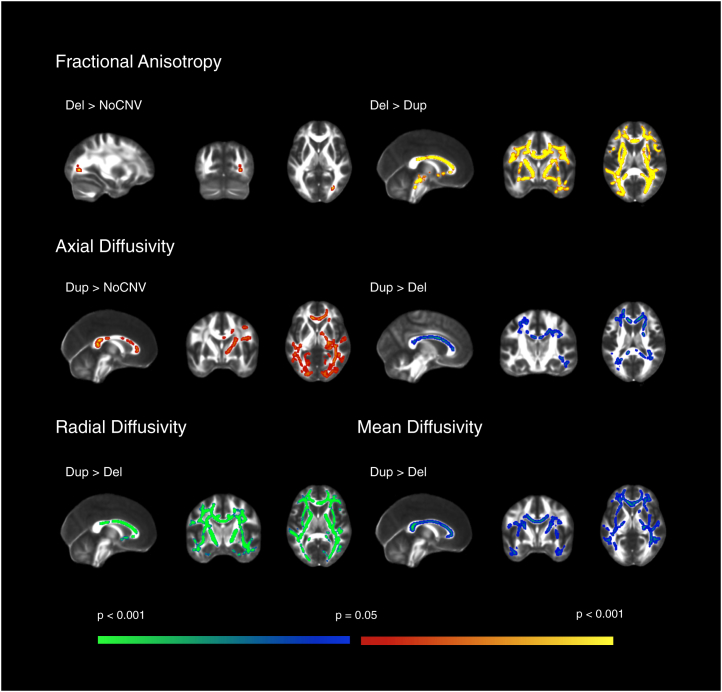
FSL Tract-Based Spatial Statistics whole-group voxel-based analysis. Significant results for the two-sample *t* test showing group differences between subjects with the deletion (Del) (*n =* 30), duplication (Dup) (*n =* 27), and no large copy number variants (NoCNV) (*n =* 19) for fractional anisotropy, axial diffusivity, radial diffusivity, and mean diffusivity maps. Here, only contrasts that gave rise to significant results after correction are displayed (*p <* .05, corrected). Within the significant results, red and blue code for less significant results and yellow and green for more significant results. The deletion showed widespread increased fractional anisotropy compared with the duplication and NoCNV groups, and decreased axial diffusivity, radial diffusivity, and mean diffusivity compared with the duplication group. The duplication group showed increased axial diffusivity compared with NoCNV group.

TBSS was used to assess groupwise microstructural differences in major WM pathways throughout the brain. *F* statistics showed extensive significant differences in the direction deletion > NoCNV > duplication in FA, and duplication > NoCNV > deletion in AD, RD, and MD. Further pairwise comparisons showed extensive and global increase in FA, and decreased AD, RD, and MD in the deletion group compared with the duplication group. These differences were seen in major WM tracts, such as the corpus callosum, superior longitudinal fasciculus, inferior longitudinal fasciculus (ILF), and internal capsule (IC). Moreover, the deletion group also showed increased FA when compared with the NoCNV group in the posterior thalamic radiation. The duplication group showed significantly increased AD when compared with the NoCNV group. The contrasts that gave rise to significant voxelwise results (*p <* .05, corrected) are summarized in Table 2 and TBSS results are displayed in Figure 1.

### Between-Group Regional Analyses

**Figure 2:**
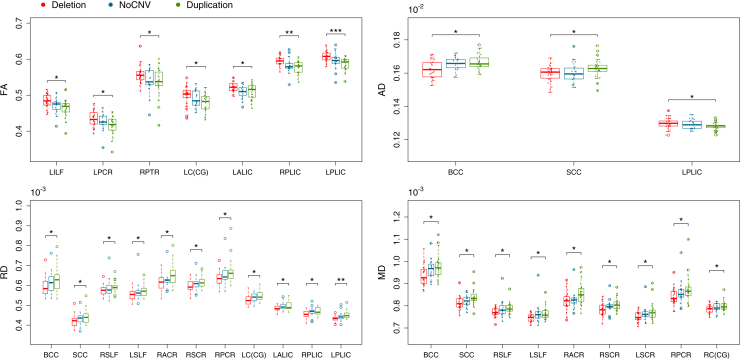
Boxplots showing group differences for atlas-based analyses. Significant group differences in fractional anisotropy (FA), axial diffusivity (AD), radial diffusivity (RD), and mean diffusivity (MD) are shown after multiple comparisons correction (*p <* .05). *p < .05, **p < .01, ***p < .001. BCC, body of corpus callosum; LALIC, left anterior limb of the internal capsule; LC(CG), left cingulum (cingulate gyrus portion); LILF, left inferior longitudinal fasciculus; LPCR, left posterior corona radiata; LPLIC, left posterior limb of the internal capsule; LSCR, left superior corona radiata; LSLF, left superior longitudinal fasciculus; NoCNV, no large copy number variants; RACR, right anterior corona radiata; RC(CG), right cingulum (cingulate gyrus portion); RPCR, right posterior corona radiata; RPLIC, right posterior limb of the internal capsule; RPTR, right posterior thalamic radiation; RSCR, right superior corona radiata; RSLF, right superior longitudinal fasciculus; SCC, splenium of the corpus callosum.

**Figure 3:**
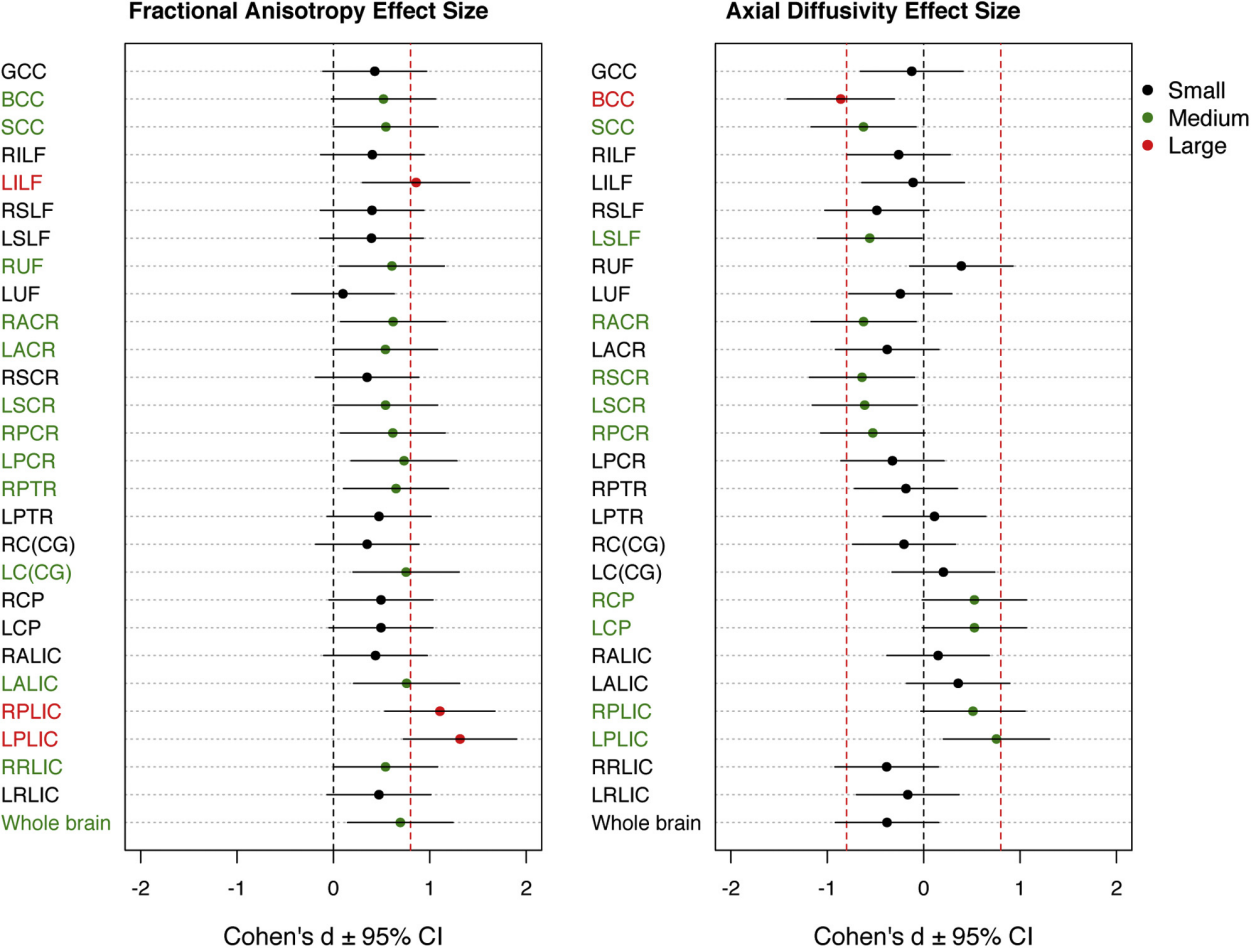
Effect sizes on deletion versus duplication for fractional anisotropy (left) and axial diffusivity (right). The threshold where an effect size is considered to be large [0.8, according to Cohen’s criteria (43)] is represented by the vertical red dashed line. Black, green, and red dots represent small, medium, and large effect sizes, respectively. BCC, body of corpus callosum; CI, confidence interval; GCC, genu of the corpus callosum; LACR, left anterior corona radiata; LALIC, left anterior limb of the internal capsule; LC(CG), left cingulum (cingulate gyrus); LCP, left cerebral peduncle; LILF, left inferior longitudinal fasciculus; LPCR, left posterior corona radiata; LPLIC, left posterior limb of the internal capsule; LPTR, left posterior thalamic radiation; LRLIC, left retrolenticular part of internal capsule; LSCR, left superior corona radiata; LSLF, left superior longitudinal fasciculus; LUF, left uncinate fasciculus; RACR, right anterior corona radiata; RALIC, right anterior limb of the internal capsule; RC(CG), right cingulum (cingulate gyrus); RCP, right cerebral peduncle; RILF, right inferior longitudinal fasciculus; RPCR, right posterior corona radiata; RPLIC, right posterior limb of the internal capsule; RPTR, right posterior thalamic radiation; RRLIC, right retrolenticular part of internal capsule; RSCR, right superior corona radiata; RSLF, right superior longitudinal fasciculus; RUF, right uncinate fasciculus; SCC, splenium of the corpus callosum.

Results from the atlas-based segmentation were consistent with the TBSS. Plots of the data confirmed the overall pattern of increased FA in the deletion group compared with the duplication group, with the NoCNV group lying intermediate between these groups (Figure 2). However, the deletion group showed greater effect sizes than the duplication group when compared with the NoCNV group (Supplemental Table S1). Because the pairwise comparisons were only significant between the deletion and duplication groups, we only show Cohen’s effect size plots for comparisons of the deletion group versus the duplication group. Cohen’s effect sizes for FA and AD are displayed in Figure 3, and for all the DTI measures in Supplemental Figure S1. The largest effect size was observed for higher FA and lower RD in the posterior limb of the IC (PLIC). Across the whole brain, the effect size was medium in FA (Cohen’s *d* = 0.69), RD (Cohen’s *d* = −0.68), and MD (Cohen’s *d* = −0.63), and small for AD (Cohen’s *d* = −0.38), according to Cohen’s criteria (43). Findings are summarized in Table 3 and extended in Supplemental Table S1. As some of the subjects are related, we reanalyzed the data using only one member from each family and found the results to be consistent with initial findings/primary analyses. However, a few WM tracts became nonsignificant, possibly owing to the loss of power from reducing the cohort to 65 subjects (Supplemental Figures S3 and S4).

**Table 3:** Comparisons Between the Deletion and Duplication of the 15q11.2 BP1-BP2 Region on FA, AD, RD, and MD

Dependent Variable	ROI	*t*	*p* Value (FDR Corrected)	Effect Size
FA Del vs. Dup	LILF	−3.15	.02^a^	0.86
	LPCR	−2.85	.03^a^	0.73
	RPTR	−2.68	.04^a^	0.65
	LC(CG)	−2.93	.03^a^	0.75
	LALIC	−2.97	.03^a^	0.76
	RPLIC	−4.31	.003^b^	1.11
	LPLIC	−5.06	.0006^c^	1.31
AD Del vs. Dup	BCC	3.55	.02^a^	−0.86
	SCC	−2.62	.04^a^	−0.62
	LPLIC	−2.88	.03^a^	0.75
RD Del vs. Dup	BCC	2.89	.03^a^	−0.65
	SCC	2.88	.03^a^	−0.68
	RSLF	2.69	.04^a^	−0.70
	LSLF	2.69	.04^a^	−0.69
	RACR	3.01	.03^a^	−0.73
	RSCR	2.78	.03^a^	−0.71
	RPCR	2.78	.03^a^	−0.69
	LC(CG)	3.22	.03^a^	−0.83
	LALIC	2.80	.03^a^	−0.72
	RPLIC	3.32	.02^a^	−0.84
	LPLIC	4.48	.002^b^	−1.16
MD Del vs. Dup	BCC	3.38	.02^a^	−0.77
	SCC	3.31	.02^a^	−0.78
	RSLF	2.99	.03^a^	−0.78
	LSLF	2.95	.03^a^	−0.77
	RACR	3.03	.03^a^	−0.76
	RSCR	2.89	.03^a^	−0.75
	LSCR	2.60	.04^a^	−0.65
	RPCR	2.63	.03^a^	−0.66
	RC(CG)	2.97	.03^a^	−0.67

AD, axial diffusivity; BCC, body of corpus callosum; Del, deletion; Dup, duplication; FA, fractional anisotropy; FDR, false discovery rate; LALIC, left anterior limb of the internal capsule; LC(CG), left cingulum (cingulate gyrus portion); LILF, left inferior longitudinal fasciculus; LPCR, left posterior corona radiata; LPLIC, left posterior limb of the internal capsule; LSCR, left superior corona radiata; LSLF, left superior longitudinal fasciculus; MD, mean diffusivity; RACR, right anterior corona radiata; RC(CG), right cingulum (cingulate gyrus portion); RD, radial diffusivity; ROI, region of interest; RPCR, right posterior corona radiata; RPLIC, right posterior limb of the internal capsule; RPTR, right posterior thalamic radiation; RSCR, right superior corona radiata; RSLF, right superior longitudinal fasciculus; SCC, splenium of the corpus callosum.

a *p* < .05.

b *p* < .01.

c *p* < .001.

### Sex Differences

A sex-by-dosage interaction model was used to investigate sex differences in relation to 15q11.2 BP1-BP2 dosage. Although we found no significant interaction effect in the whole-group analysis, we found significant differences in effect size when analyzing men and women separately, as assessed by using a two-tailed unpaired *t* test. Men showed larger effect size for increased FA (*t =* 2.56, *p =* .013) compared with women, and an overall large effect size in the whole brain (Cohen’s *d* = 0.99), whereas women showed a small effect size (Cohen’s *d* = 0.47). Moreover, men showed large effect sizes in more regions, namely in the genu and body of the corpus callosum, left ILF, anterior and posterior corona radiata, posterior thalamic radiation, cerebral peduncle, anterior limb of the IC, and PLIC. Women, however, showed a large effect size for increased FA in the left cingulum (cingulate gyrus portion) that is not seen in men (Supplemental Figure S2).

## Discussion

In a whole-brain exploratory analysis, we found consistently increased FA and decreased RD and MD in individuals with the 15q11.2 BP1-BP2 deletion compared with individuals in the reciprocal duplication group. The duplication group showed significantly increased AD relative to the NoCNV and deletion groups (Figure 1). Additional regional analyses (Figure 2) indicated that, in most WM tracts, the NoCNV group was intermediate between the deletion and duplication groups, suggesting a “mirror phenotype” (12). However, the deletion showed a greater impact on WM microstructure by showing larger effect sizes than in the duplication group (Supplemental Table S1).

We found the greatest effects in FA and RD bilaterally in the PLIC (Figure 2 and Supplemental Figure S1). The PLIC carries sensory information from the thalamus to the cortex, a key sensorimotor relay area implicated in schizophrenia (44) and ASD (45). In schizophrenia, reductions in FA have been reported in the IC (4). However, in ASD patients, functional connectivity between motor regions of the thalamus and cortex was found to be hyperconnected (46), and a longitudinal study showed that the thalamus and IC undergo an atypical development trajectory in ASD, in which increasing connectivity from childhood through adolescence and adulthood was seen (47). The increased FA in the PLIC seen in the deletion group could be a result of an abnormal thalamus and IC development, which could relate to motor delays frequently reported in the BP1-BP2 deletion. Thus, a younger group is needed to look at the age trajectory of FA and its correlates with motor function outcome. We also found a large effect size in FA in the left ILF, a major WM tract thought to be critical to semantic processing and involved in dyslexia. Dyslexia and dyscalculia are common features in 15q11.2 BP1-BP2 deletion, and individuals with the deletion were previously shown to have a smaller fusiform gyrus (13), a structure that was shown to play a role in reading and mathematics and that connects to the ILF (48).

Although all DTI changes seem to be consistent throughout the brain, regional analysis shows increases and decreases in AD in different WM tracts in the deletion (Figure 2, Figure 3). Previously, AD has been related to axonal damage and RD with axonal density and myelin (49). FA reflects the relative contribution of AD and RD. Because we find global increased FA, including areas where AD is decreased, the RD contribution seems to be stronger. The global decreased RD in the corpus callosum (and other areas) found here could be a result of increased axonal density that may also explain the increased WM volume found previously in the corpus callosum in healthy individuals with the deletion 12, 13. Furthermore, areas with reduced AD could be a result of reduced axonal integrity.

Increased FA arising from abnormal WM organization has been reported before in patients with Williams syndrome, a chromosomal disorder associated with visuospatial deficits, in which higher FA in the superior longitudinal fasciculus tract was correlated with deficits in visuospatial construction (50). The globally increased FA in the deletion group could point to either a compensatory mechanism in response to primary deficits, as a protection against disease onset, or a diffuse dysregulation of neuronal dynamics, increasing the risk for psychiatric disorder. Hence, a central question is how each gene within this CNV region could contribute to this phenotype. All four genes in this region are highly conserved and expressed in human central nervous system, and may play a role in 15q11.2 BP1-BP2–associated phenotypes. Moreover, mutations in each gene were associated with different disorders: *NIPA1* with autosomal-dominant hereditary spastic paraplegia (17), *NIPA2* with childhood absence epilepsy (19), *TUBGCP5* with ADHD and obsessive-compulsive disorder (20), and *CYFIP1* with increasing susceptibility to ASD (51) and with schizophrenia (52). Furthermore, dysregulations in mechanisms related to *NIPA1* and *CYFIP1* genes might have an impact on WM microstructure. *NIPA1* was found to inhibit the BMP signaling via interaction with BMP receptor type II (18), which is crucial for typical axonal growth, guidance, and differentiation. In a *Drosophila* model, enhanced BMP signaling resulted in abnormal distal axonal overgrowth at the presynaptic neuromuscular junction (53), which could result in increased axonal density. *CYFIP1*, on the other hand, has a crucial role in actin remodeling during neural wiring, in which dysregulations could result in changes in axonal density, organization, and myelination 54, 55.

Recently, two articles by the same group [Green *et al.* (27) and Hall *et al.* (28)] reported increased FA and decreased RD and MD in FXS patients compared with IQ-matched control subjects. There is, therefore, a marked degree of overlap between our current findings in 15q11.2 BP1-BP2 deletion and WM changes in FXS, consistent a priori with the suggested molecular link between CYFIP1 and FMRP. The question arises, what common neural mechanism(s) may contribute to this overlap in WM phenotype? Here, evidence that both FMRP and CYFIP1 influence diverse aspects of synaptic function, as well as effects on dendritic architecture, may be of relevance 56, 57, 58. Both *Fmr1* knockout and *Cyfip1* hemizygous-null adult mice have in common an increased ratio of immature-to-mature spines 59, 60, 61, 62, 63. While the relationship between neuronal density and number of synapses per neuron is still not well understood, the observed increased FA in FXS 26, 27 and 15q11.2 BP1-BP2 deletion (this study) could be caused by an increased neuronal density as an adaptive response to an increased number of immature spines and reduced functional synapses.

Further speculations as to cellular/molecular mechanisms underlying the observed WM changes should, at this stage, be made with caution. DTI data are difficult to relate in a definitive way to underlying cellular changes, and their investigation would require postmortem or biopsy. To overcome this, translational models of human disease in animals are an attractive alternative to explore individual genotype-phenotype relationships (64). Therefore, owing to the potential role of *CYFIP1* in WM microstructure phenotypes associated with the 15q11.2 BP1-BP2 region, it would be informative to assess DTI data using low-dosage *Cyfip1* animal models. Furthermore, direct access to brain tissue would allow an analysis of underlying cellular changes relevant to the DTI findings.

Clinical phenotypes of reciprocal CNVs have been broadly classified into four general categories: mirrored, identical, overlapping, and unique (65). The 16p11.2 (66), 1q21.1 (67), 3q29 (68), and 17p11.2 (69) CNVs have been associated with mirrored phenotypes. Comparable to what we have reported here, increased FA was found in individuals with the 16p11.2 deletion, and opposite changes were found in individuals with the reciprocal duplication (70). The extensive reciprocal effects on WM reported here, and in previous studies 12, 13, show that the 15q11.2 BP1-BP2 also affects WM microstructure in a dosage-dependent way. When it comes to neuropsychiatric and behavioral findings at this locus, the picture is less clear (6). The microdeletion has been associated with developmental delay, schizophrenia, and autism, whereas duplication is generally not considered as a risk locus for schizophrenia (71) and has not come out as a significant risk variant for developmental delay in recent large-scale genetic studies (72). Moreover, the microdeletion has been shown to have a greater impact on cognitive function in healthy individuals, particularly in the acquisition of mathematical skills and reading, whereas individuals in the duplication group performed similarly to the NoCNV group 12, 13. In this study, the microdeletion also shows a greater impact on WM microstructure, with larger effect sizes than the microduplication (Supplemental Table S1), but the lack of cognitive data in this sample did not allow us to find correlations between increased FA and cognition.

A limitation of this study was the impossibility to correct regions with crossing fibers, and reductions in the number of fibers in these regions might give rise to increased FA. The fact that we see an overall increased FA, and not only in crossing fiber regions, makes this less likely to be the main cause of the group differences. In the current analysis, we could not find a sex-by-dosage interaction, but men showed larger effect sizes than women (Supplemental Figure S2), suggesting sex-dependent changes in WM. Although the molecular causality behind this sex difference is still unclear, sex bias has been observed in neurodevelopmental disorders (73). Moreover, 15q11.2 BP1-BP2 was shown to have a greater impact on ASD-related phenotype in men than women (8). Further larger studies will, however, be required to determine the exact interaction of sex and 15q11.2 BP1-BP2 dosage.

Using complementary methods of analysis, this study shows a consistent pattern of WM microstructure alterations, which are consistent with recent FXS DTI studies, beginning to reveal brain mechanisms underlying the complex routes to psychopathology mediated by mutations at the 15q11.2 BP1-BP2 cytogenetic region. The reciprocal effects on WM microstructure, described here, suggest that deviations from normal gene dosage in each direction can lead to abnormalities in brain development, underlining the importance of studying how reciprocal chromosomal imbalances impact neural processes, which might have important implications for therapeutic intervention.
