# Supplementary material for: Reciprocal White Matter Changes Associated With Copy Number Variation at 15q11.2 BP1-BP2: A Diffusion Tensor Imaging Study
Source: Biol Psychiatry. 2019 Apr 1;85(7):563–72. doi: 10.1016/j.biopsych.2018.11.004 (PMC6424871; doi:10.1016/j.biopsych.2018.11.004)
Supplement: Supplemental Material [file mmc1.pdf]

Reciprocal White Matter Changes Associated With Copy Number Variation at 15q11.2 BP1-BP2: A Diffusion Tensor Imaging Study

Supplemental Information

Methods

Diffusion Tensor Imaging Measures

Changes in axonal diameter, density, cytoskeletal properties, swelling or neuroinflammation can affect the degree of anisotropy of the water molecules. DTI findings are commonly reported in terms of scalars such as fractional anisotropy (FA), radial diffusivity (RD), axial diffusivity (AD), and mean diffusivity (MD). FA describes the degree of anisotropy of a diffusion process and correlates with higher density, diameter, degree of organization and myelination of axons. AD is the measure of water diffusivity along the main axis of diffusion, and is thought to reflect fiber coherence. RD is characterized by the perpendicular diffusivities to the main axis of diffusion, and is inversely correlated with myelin thickness and fiber density. Lastly, MD is the average diffusion across all directions.

Table S1. Effect of 15q11.2 BP1-BP2 dosage on fractional anisotropy (FA), axial diffusivity (AD), radial diffusivity (RD), and mean diffusivity (MD).

	Dependent variable	ROI	Coefficient value	SE	*p* Value (FDR corrected)	Effect size
FA	Del vs NoCNV	LILF	-1.53E-02	6.43E-03	0.38	0.64
		LPCR	-7.01E-03	7.42E-03	0.71	0.40
		RPTR	-1.52E-02	8.19E-03	0.87	0.59
		LC(CG)	-1.26E-02	8.04E-03	0.63	0.36
		LALIC	-1.28E-02	5.28E-03	0.39	0.88
		RPLIC	-1.22E-02	5.14E-03	0.39	0.82
		LPLIC	-8.75E-03	5.56E-03	0.63	0.62
	Dup vs NoCNV	LILF	-4.63E-03	7.67E-03	0.78	-0.26
		LPCR	-7.58E-03	8.03E-03	0.78	-0.34
		RPTR	-3.17E-03	1.18E-02	0.88	-0.11
		LC(CG)	-7.88E-03	7.86E-03	0.78	-0.41
		LALIC	3.53E-04	6.55E-03	0.98	0.04
		RPLIC	-3.71E-03	5.90E-03	0.78	-0.17
		LPLIC	-8.46E-03	6.01E-03	0.78	-0.43
	Del vs Dup	LILF	-2.109E-02	6.01E-03	0.02*	0.86
		LPCR	-1.885E-02	6.61E-03	0.03*	0.73
		RPTR	-2.28E-02	8.50E-03	0.04*	0.65
		LC(CG)	-2.02E-02	6.88E-03	0.03*	0.75
		LALIC	-1.45E-03	4.88E-03	0.03*	0.76
		RPLIC	-1.82E-03	5.23E-03	0.003**	1.11
		LPLIC	-2.13E-02	2.206E-03	0.0006***	1.31
AD	Del vs NoCNV	BCC	3.64E-05	1.51E-05	0.39	-0.71
		SCC	4.44E-06	1.69E-05	0.99	-0.10
		LPLIC	-8.11E-06	8.59E-06	0.71	0.19
	Dup vs NoCNV	BCC	9.19E-06	1.39E-05	0.78	0.21
		SCC	4.06E-05	1.67E-05	0.41	0.44
		LPLIC	-1.116E-05	7.04E-06	0.78	-0.57
	Del vs Dup	BCC	4.70E-05	1.33E-05	0.02*	-0.86
		SCC	3.49E-05	1.33E-05	0.04*	-0.62
		LPLIC	-1.95E-05	6.79E-06	0.03*	0.75
RD	Del vs NoCNV	BCC	2.33E-05	1.56E-05	0.63	-0.45
		SCC	6.35E-06	9.55E-06	0.82	-0.38
		RSLF	5.26E-06	1.06E-05	0.87	-0.34
		LSLF	8.55E-06	1.14E-05	0.77	-0.45
		RACR	3.54E-06	1.30E-05	0.99	-0.18
		RSCR	8.95E-06	9.02E-06	0.71	-0.41
		RPCR	1.16E-05	1.38E-05	0.73	-0.40
		LC(CG)	9.59E-06	8.05E-06	0.69	-0.49
		LALIC	7.73E-06	5.47E-06	0.71	-0.63
		RPLIC	1.02E-05	6.21E-06	0.63	-0.70
		LPLIC	7.86E-06	5.52E-06	0.63	-0.66
	Dup vs NoCNV	BCC	1.35E-05	1.79E-05	0.78	0.23
		SCC	1.66E-05	1.06E-05	0.78	0.31
		RSLF	1.04E-05	1.09E-05	0.78	0.14
		LSLF	7.58E-06	1.24E-05	0.78	0.06
		RACR	2.99E-05	1.51E-05	0.63	0.60
		RSCR	9.28E-06	9.91E-06	0.78	0.24
		RPCR	1.66E-05	1.83E-05	0.78	0.27
		LC(CG)	1.07E-05	8.07E-06	0.78	0.37
		LALIC	3.27E-06	7.05E-06	0.81	0.13
		RPLIC	4.87E-06	6.55E-06	0.78	0.08
		LPLIC	7.14E-06	6.45E-06	0.78	0.34

	Dependent variable	ROI	Coefficient value	SE	*p* Value (FDR corrected)	Effect size
		BCC	4.14E-05	1.43E-05	0.03*	-0.65
		SCC	2.48E-05	8.62E-06	0.03*	-0.68
		RSLF	1.84E-05	6.84E-06	0.04*	-0.70
		LSLF	2.07E-05	7.71E-06	0.04*	-0.69
		RACR	1.94E-05	6.43E-06	0.03*	-0.73
	Del vs Dup	RSCR	2.10E-05	7.57E-06	0.03*	-0.71
		RPCR	3.62E-05	1.30E-05	0.03*	-0.69
		LC(CG)	2.21E-05	6.88E-06	0.03*	-0.83
		LALIC	1.67E-05	5.03E-06	0.03*	-0.72
		RPLIC	8.35E-06	2.52E-06	0.02*	-0.84
		LPLIC	2.01E-05	4.48E-06	0.002**	-1.16
		BCC	2.77E-05	1.43E-05	0.51	-0.58
		SCC	5.71E-06	9.10E-06	0.85	-0.33
		RSLF	7.82E-06	8.62E-06	0.71	-0.54
		LSLF	1.10E-05	9.55E-06	0.70	-0.62
	Del vs NoCNV	RACR	2.19E-06	1.23E-05	0.99	-0.13
		RSCR	1.17E-05	8.74E-06	0.66	-0.54
		LSCR	7.20E-06	8.27E-06	0.71	-0.49
		RPCR	1.37E-05	1.39E-05	0.71	-0.45
		RC(CG)	6.33E-07	5.99E-06	0.99	-0.32
		BCC	1.20E-05	1.54E-05	0.78	0.24
		SCC	2.46E-05	9.86E-06	0.41	0.50
MD		RSLF	7.27E-06	9.90E-06	0.78	0.04
		LSLF	3.67E-06	1.15E-05	0.86	-0.04
	Dup vs NoCNV	RACR	3.18E-05	1.42E-05	0.42	0.66
		RSCR	9.37E-06	9.40E-06	0.78	0.21
		LSCR	9.45E-06	1.22E-05	0.78	0.21
		RPCR	1.79E-05	1.83E-05	0.78	0.22
		RC(CG)	1.19E-05	6.69E-06	0.74	0.36
		BCC	4.33E-05	1.28E-05	0.02*	-0.77
		SCC	1.41E-05	4.25E-06	0.02*	-0.78
		RSLF	1.82E-05	6.09E-06	0.03*	-0.78
		LSLF	1.97E-05	6.68E-06	0.03*	-0.77
	Del vs Dup	RACR	3.68E-05	1.21E-05	0.03*	-0.76
		RSCR	2.27E-05	7.87E-06	0.03*	-0.75
		LSCR	2.28E-05	8.77E-06	0.04*	-0.65
		RPCR	3.60E-05	1.37E-05	0.03*	-0.66
		RC(CG)	1.19E-05	6.69E-06	0.03*	-0.67

Abbreviations used: body, and splenium of the corpus callosum (BCC, SCC), left inferior longitudinal fasciculus (LILF), right and left superior longitudinal fasciculus (RSLF, LSLF), right anterior corona radiata (RACR), right and left superior corona radiata (RSCR, LSCR), right and left posterior corona radiata (RPCR, LPCR), right posterior thalamic radiation (RPTR), right and left cingulum (cingulate gyrus portion) (RC(CG), LC(CG)), left anterior limb of the internal capsule (LALIC), right and left posterior limb of the internal capsule (RPLIC, LPLIC). (* < 0.05, ** < 0.01, ***<0.001).

Table S2. Individual information on dosage, age, gender, total intracranial volume, and family relationships. Individuals who are related are highlighted with a certain color, and the color code is specified in the KEY column. The individuals who were removed from the analyses to produce Figures S3 and S4 are specified on column 6.

Subject	dosage	age	gender	TIV	
Subj 1	2	59	1	1612.33	
Subj 2	2	41	1	1767.58	removed
Subj 3	2	37	1	1642.45	
Subj 4	2	49	2	1500.51	
Subj 5	2	56	1	1916.71	
Subj 6	2	36	2	1329.03	
Subj 7	2	22	1	1640.82	
Subj 8	2	42	1	1860.66	
Subj 9	2	39	2	1629.37	
Subj 10	2	26	2	1638.81	
Subj 11	2	31	2	1420.73	
Subj 12	2	37	1	1569.75	
Subj 13	2	28	2	1486.33	
Subj 14	3	62	1	1769.55	
Subj 15	3	45	1	1616.38	
Subj 16	1	44	2	1575.26	
Subj 17	1	32	2	1376.6	
Subj 18	1	27	1	1558.38	
Subj 19	1	56	2	1385.12	
Subj 20	1	38	1	1544.59	
Subj 21	1	27	1	1860.96	
Subj 22	1	41	2	1479.42	
Subj 23	3	52	2	1546.55	removed
Subj 24	1	65	1	1547.32	
Subj 25	1	45	2	1458.81	
Subj 26	3	26	2	1333.78	removed
Subj 27	3	53	1	1715.04	
Subj 28	3	51	2	1484.94	removed
Subj 29	2	35	1	1701.68	
Subj 30	1	24	1	1662.12	
Subj 31	3	58	2	1382.59	
Subj 32	1	27	1	1593.04	removed
Subj 33	1	37	2	1543.53	
Subj 34	1	23	2	1414.08	removed
Subj 35	3	40	2	1509.29	
Subj 36	1	57	2	1416.03	
Subj 37	3	26	2	1464.85	

KEY

(yellow)	nephew-aunt
(orange)	half-siblings
(light green)	mother-daughter
(light blue)	siblings
(dark blue)	siblings
(light orange)	daughter-father
(grey)	son-mother
(red)	siblings
(cyan)	uncle-niece
(purple)	first cousins
(pink)	first cousins
1	Deletion
2	NoCNV
3	Duplication

Subject	dosage	age	gender	TIV	
Subj 38	3	49	1	1499.49	
Subj 39	3	28	1	1676.73	
Subj 40	2	25	1	1791.84	
Subj 41	2	50	1	1676.31	
Subj 42	3	22	2	1607.84	
Subj 43	3	51	1	1552.31	
Subj 44	1	46	2	1589.06	
Subj 45	3	54	1	1706.39	removed
Subj 46	1	50	1	1692.26	
Subj 47	3	23	1	1770.81	
Subj 48	1	56	1	1426.4	
Subj 49	1	60	2	1505.46	
Subj 50	3	22	1	1607.08	
Subj 51	1	61	1	1643.26	
Subj 52	3	59	2	1530.1	removed
Subj 53	1	50	1	1526.48	
Subj 54	3	51	2	1458.54	
Subj 55	3	41	1	1725.68	
Subj 56	3	35	2	1467.06	
Subj 57	3	27	2	1547.91	
Subj 58	1	55	1	1749.57	
Subj 59	3	48	2	1412.99	
Subj 60	1	54	2	1328.75	removed
Subj 61	2	35	2	1295.43	
Subj 62	3	57	1	1551.93	
Subj 63	3	37	2	1379.58	
Subj 64	3	36	2	1506.7	
Subj 65	1	34	1	1630.99	
Subj 66	1	42	2	1465.2	
Subj 67	1	47	2	1272.6	removed
Subj 68	3	65	2	1418.19	
Subj 69	1	40	2	1475.89	
Subj 70	1	21	1	1675.78	
Subj 71	1	53	1	1449.1	
Subj 72	1	32	2	1447.54	
Subj 73	1	41	2	1370.07	removed
Subj 74	3	56	1	1638.58	
Subj 75	2	53	1	1821.95	
Subj 76	2	39	1	1700.44	

KEY

Colour	Relationship
yellow	nephew-aunt
orange	half-siblings
light green	mother-daughter
light blue	siblings
dark blue	siblings
light yellow	daughter-father
grey	son-mother
red	siblings
cyan	uncle-niece
purple	first cousins
magenta	first cousins

1	Deletion
2	NoCNV
3	Duplication

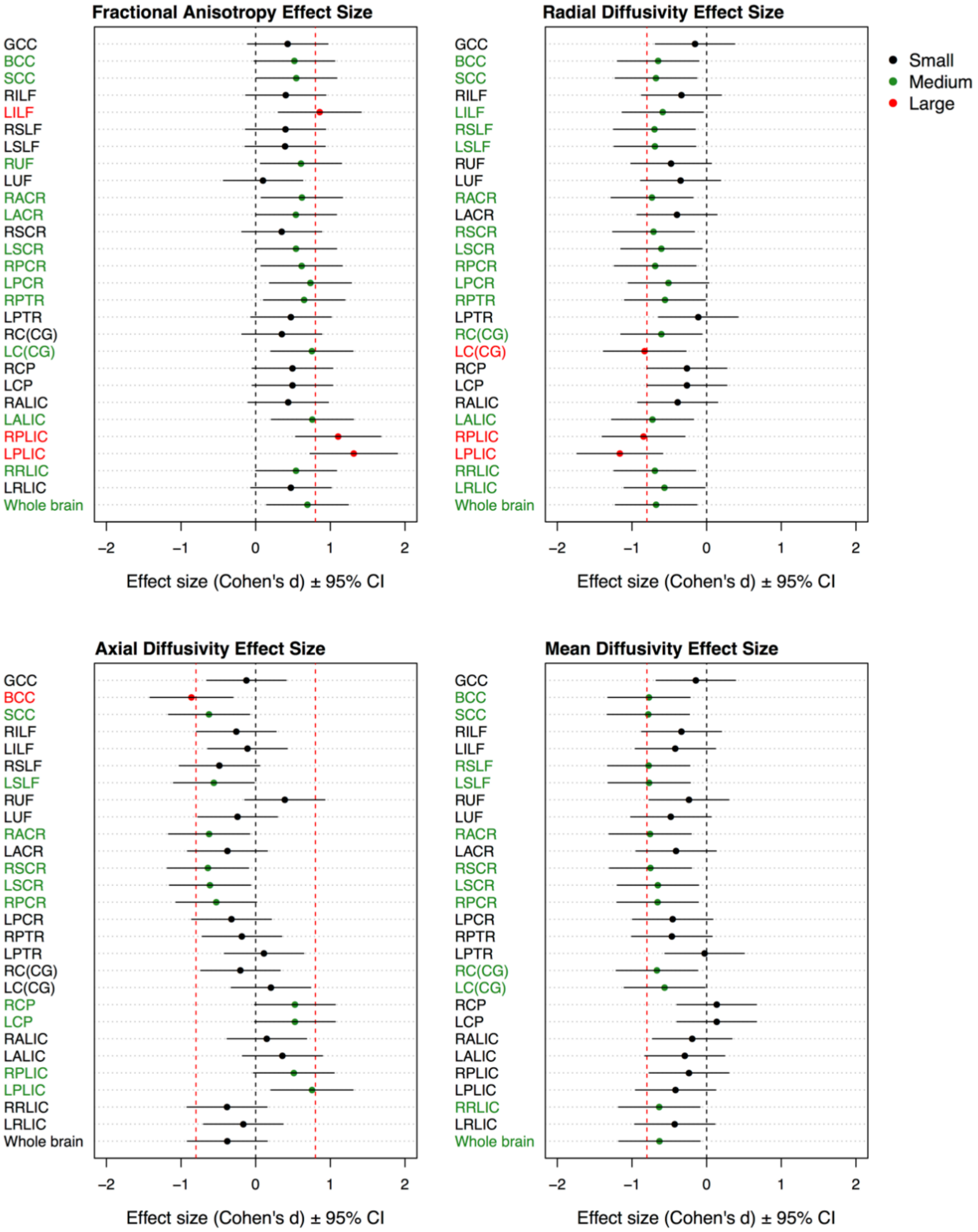

Figure S1. Effect sizes on deletion versus duplication for fractional anisotropy, radial diffusivity, axial diffusivity and mean diffusivity. The threshold where an effect size is considered to be large (0.8, according to Cohen's criteria (1)) is represented by the vertical red dashed line. Black, green and red dots represent small, medium and large effect sizes respectively. **Abbreviations used:** genu, body and splenium of the corpus callosum (GCC, BCC, SCC), right and left inferior longitudinal fasciculus (RILF, LILF), right and left superior longitudinal fasciculus (RSLF, LSLF), right and left uncinate fasciculus (RUF, LUF), right and left anterior corona radiata (RACR, LACR), right and left superior corona radiata (RSCR, LSCR), right and left posterior corona radiata (RPCR, LPCR), right and left posterior thalamic radiation (RPTR, LPTR), right and left cingulum (cingulate gyrus) (RC(CG), LC(CG)), right and left cerebral peduncle (RCP, LCP), right and left anterior limb of the internal capsule (RALIC, LALIC), right and left posterior limb of the internal capsule (RPLIC, LPLIC), right and left retrolenticular part of internal capsule (RRLIC, LRLIC).

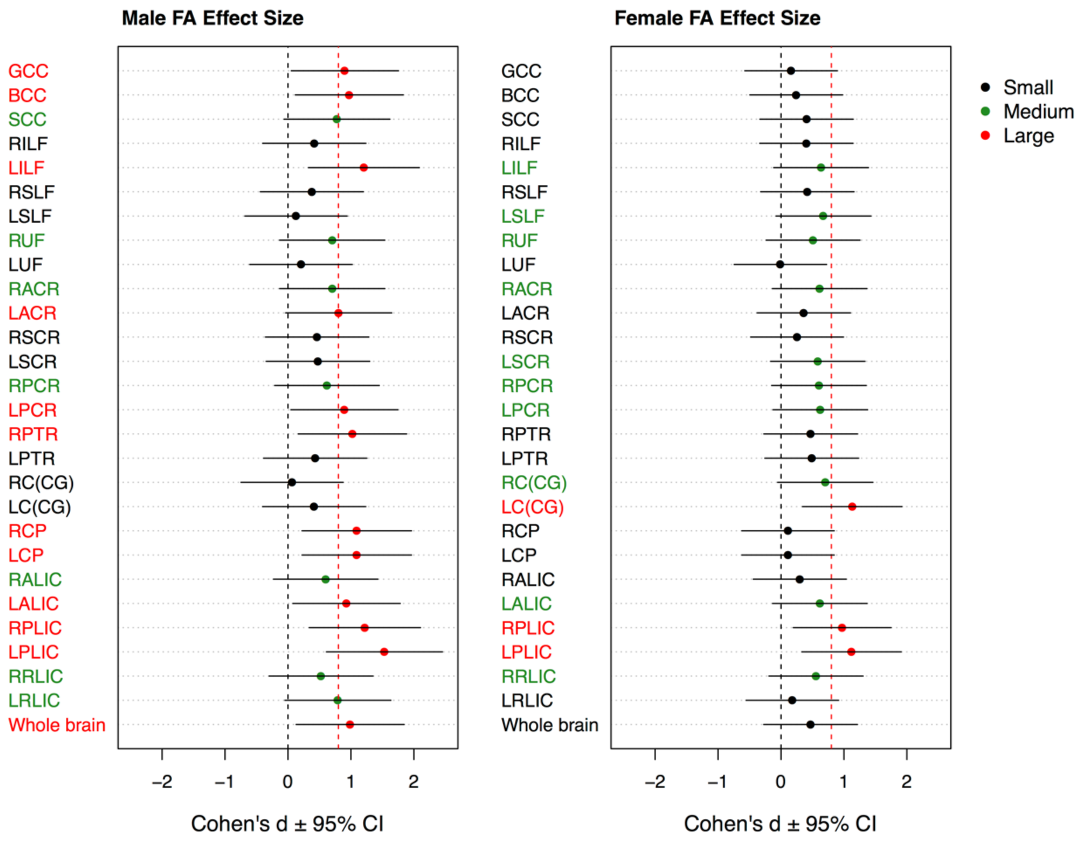

Figure S2. Effect sizes on deletion versus duplication for fractional anisotropy for males only (left) and females only (right). The threshold where an effect size is considered to be large (0.8, according to Cohen's criteria (1)) is represented by the vertical red dashed line. Black, green and red dots represent small, medium and large effect sizes respectively**. Abbreviations used:** genu, body and splenium of the corpus callosum (GCC, BCC, SCC), right and left inferior longitudinal fasciculus (RILF, LILF), right and left superior longitudinal fasciculus (RSLF, LSLF), right and left uncinate fasciculus (RUF, LUF), right and left anterior corona radiate (RACR, LACR), right and left superior corona radiate (RSCR, LSCR), right and left posterior corona radiata (RPCR, LPCR), right and left posterior thalamic radiation (RPTR, LPTR), right and left cingulum (cingulate gyrus) (RC(CG), LC(CG)), right and left cerebral peduncle (RCP, LCP), right and left anterior limb of the internal capsule (RALIC, LALIC), right and left posterior limb of the internal capsule (RPLIC, LPLIC), right and left retrolenticular part of internal capsule (RRLIC, LRLIC).

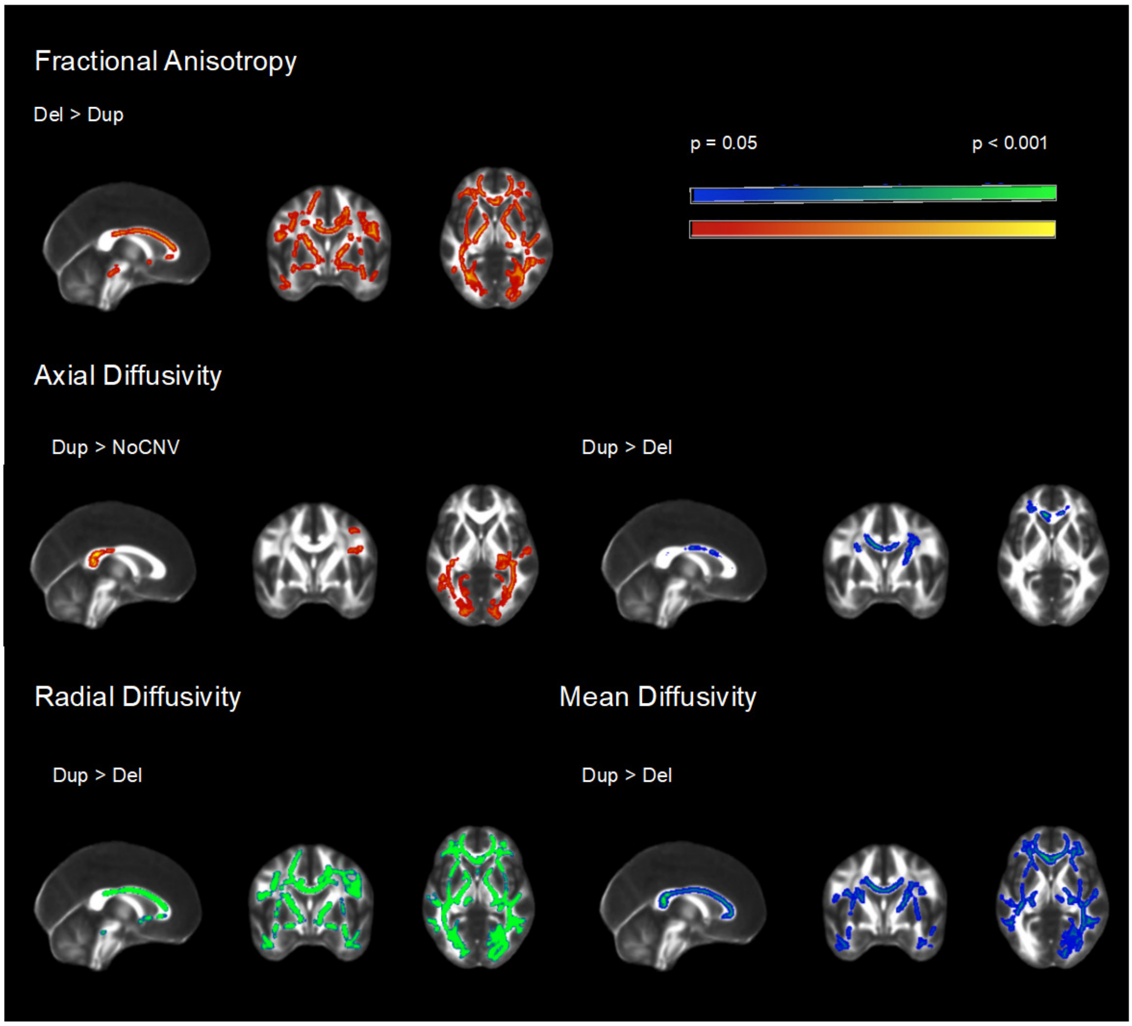

Figure S3. TBSS whole group voxel-based analysis excluding individuals who are related. Significant results for the two-sample t-test showing group differences between deletion (Del, n = 25), duplication (Dup, n = 22), and no large copy number variants (NoCNV, n = 18) groups for fractional anisotropy (FA), axial diffusivity (AD), radial diffusivity (RD), and mean diffusivity (MD) maps. Here, only contrasts that gave rise to significant results after correction are displayed ($p<0.05$, corrected). Within the significant results, red and blue code for less significant results and yellow and green for more significant results. The deletion showed widespread increased FA, and decreased AD, RD, and MD compared with duplication group. The duplication showed increased AD compared with NoCNV group.

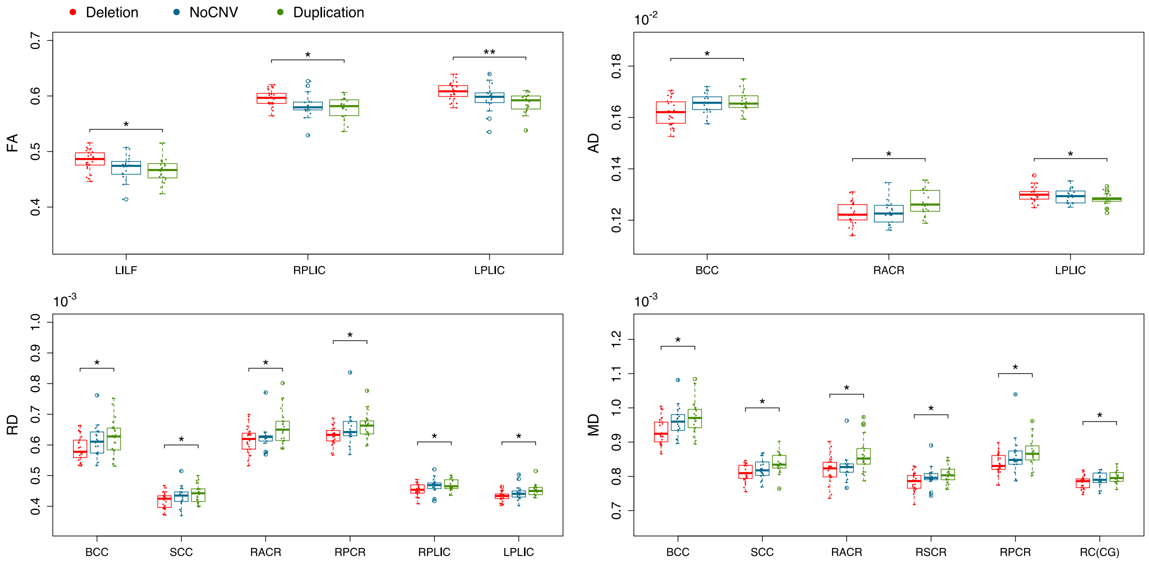

Figure S4. Boxplots showing group differences for atlas-based analyses excluding individuals who are related. Significant group differences in fractional anisotropy (FA), axial diffusivity (AD), radial diffusivity (RD), and mean diffusivity (MD) are shown after multiple comparisons correction ($p<0.05$). **Abbreviations used:** body, and splenium of the corpus callosum (BCC, SCC), left inferior longitudinal fasciculus (LILF), right anterior corona radiata (RACR), right superior corona radiata (RSCR), right posterior corona radiata (RPCR), right cingulum (cingulate gyrus portion) (RC(CG)), right and left posterior limb of the internal capsule (RPLIC, LPLIC). (* < 0.05, ** < 0.01, ***<0.001).

Supplemental Reference

1. Cohen J. CHAPTER 1 - The Concepts of Power Analysis. In: Statistical Power Analysis for the Behavioral Sciences (Revised Edition) [Internet]. Academic Press; 1977. p. 1–17. Available from: https://www.sciencedirect.com/science/article/pii/B9780121790608500062.
